# Drug transporters: recent advances concerning BCRP and tyrosine kinase inhibitors

**DOI:** 10.1038/sj.bjc.6604213

**Published:** 2008-02-05

**Authors:** C Lemos, G Jansen, G J Peters

**Affiliations:** 1 Department of Medical Oncology, VU University Medical Center, Amsterdam, The Netherlands; 2 Department of Biochemistry (U38-FCT), Faculty of Medicine, University of Porto, Porto, Portugal; 3 Department of Rheumatology, VU University Medical Center, Amsterdam, The Netherlands

**Keywords:** BCRP, multidrug resistance, polymorphisms, stem cells, targeted therapy

## Abstract

Multidrug resistance is often associated with the (over)expression of drug efflux transporters of the ATP-binding cassette (ABC) protein family. This minireview discusses the role of one selected ABC-transporter family member, the breast cancer resistance protein (BCRP/ABCG2), in the (pre)clinical efficacy of novel experimental anticancer drugs, in particular tyrosine kinase inhibitors.

Cellular drug resistance is a major obstacle in cancer therapy. Cancer cells can acquire resistance to a single drug, to a class of cytotoxic or novel targeted drugs or to a broad spectrum of structurally and functionally unrelated drugs. This latter phenomenon is known as multidrug resistance (MDR) and can be conveyed by several mechanisms including reduced drug uptake, activation of DNA repair and defective apoptotic pathways. Most commonly, however, MDR results from the active, ATP-dependent transport of drugs out of the cell by efflux pumps belonging to the ATP-binding cassette (ABC) family of transporters (Borst and Elferink, 2002). For more than 30 years, P-glycoprotein (P-gp/ABCB1) has been the most studied MDR transporter (for a comprehensive review, see Gottesman and Ling, 2006). Nowadays, it is established that also other ABC transporters such as the multidrug resistance protein 1 (MRP1/ABCC1) (Deeley *et al*, 2006; Hooijberg *et al*, 2006) and the breast cancer resistance protein (BCRP/ABCG2) (Sarkadi *et al*, 2006) are involved in MDR. This minireview highlights some recent and clinically relevant aspects of the BCRP drug efflux transporter from the perspective of current cancer chemotherapy, in particular targeted therapy with tyrosine kinase inhibitors (TKIs).

## BCRP

Breast cancer resistance protein is a half transporter member of the ABCG subfamily (ABCG2) and was first identified by Doyle *et al* (1998) in a human breast cancer cell line selected for doxorubicin resistance in the presence of verapamil, an inhibitor of P-gp. Overexpression of BCRP is associated with resistance to a wide range of different anticancer agents including mitoxantrone, camptothecins, anthracyclines, flavopiridol and antifolates (Table 1) (Assaraf, 2006; Robey *et al*, 2007). More recently, BCRP has also been shown to confer resistance to some purine analogues such as 9-(2-phosphonylmethoxyethyl)adenine and cladribine (Takenaka *et al*, 2007).

Several studies have been dedicated to determine relationships between the expression of BCRP in human cancers and clinical outcome for several drugs (summarised in Robey *et al*, 2007). Expression of BCRP has been reported in different types of leukaemia (AML and ALL) and solid tumours. The large variety of tumour types may have led to a lack of consensus regarding the impact of BCRP expression on clinical outcome, as some groups reported a correlation between BCRP expression and poor treatment response while others found no correlation. Furthermore, different treatment regimens were used for the various malignancies, adding another complicating factor. It is currently being appreciated that several additional factors should be considered to allow more accurate assessments of the role of BCRP in drug resistance and clinical outcome. These may include (a) genetic polymorphisms of BCRP that may affect substrate specificity and hence cross-resistance patterns, (b) regulation of BCRP expression by intracellular phosphorylation and/or BCRP promoter methylation and (c) a role for BCRP in the gut determining bioavailability of several drugs (Robey *et al*, 2007). The following sections in this review focus on these aspects in the context of deciphering the role of BCRP in the efficacy of a specific class of targeted agents, that is, TKIs.

## TKIS

It has been described that several TKIs are able to interact with members of the ABC family of transporters such as BCRP, P-gp and MRP1 (Hegedus *et al*, 2002; Shukla *et al*, 2007). Most studies have been focused on the interaction between TKIs and BCRP, while scarce information is available for the interaction of MRP1 or other MRPs and these drugs. However, since several controversial findings have been published regarding this subject, we set out to analyse and discuss some recent data and to further clarify potential interaction(s) between BCRP and TKIs.

### Canertinib

Canertinib (CI-1033) is a HER family TKI that has been shown to interact with BCRP. Erlichman *et al* (2001) showed that MDA-MB-231 cells transfected with BCRP had 4.9-fold lower accumulation of canertinib than cells transfected with empty vector, suggesting that canertinib is a substrate for BCRP. In both BCRP-transfected cells and unselected HCT8 colorectal carcinoma and T98G glioblastoma cells with endogenous BCRP expression, canertinib sensitised cells to SN-38 and topotecan. Consistently, canertinib increased the cellular accumulation of these drugs (Erlichman *et al*, 2001).

### Imatinib

Imatinib mesylate is a TKI of BCR-ABL, platelet-derived growth factor receptor and stem cell factor/c-kit. Conflicting results have been published regarding the ability of BCRP to transport this compound. In one study, overexpression of BCRP in Saos2 cells did not confer resistance to imatinib, and accumulation and efflux of this drug were not affected by BCRP expression and ATP depletion (Houghton *et al*, 2004), suggesting that imatinib is not a substrate of BCRP. Rather, imatinib may serve as a potent BCRP inhibitor thereby enabling reversal of BCRP-mediated resistance to topotecan and SN-38 (Houghton *et al*, 2004). Similarly, accumulation of mitoxantrone in primary chronic myeloid leukaemia (CML) CD34+ cells overexpressing BCRP was significantly increased by 5 *μ*M of imatinib, confirming the activity of this TKI as a BCRP inhibitor (Jordanides *et al*, 2006). The same authors postulated that imatinib is not a BCRP substrate, since BCRP inhibition in CML CD34+ cells neither potentiated the effect of imatinib nor affected imatinib accumulation in these cells. In contrast, Burger *et al* (2004) showed that MCF7/MR and MCF7/AdVp3000 cells, with overexpression of BCRP, had significantly lower intracellular imatinib accumulation compared with the parental MCF7 cell line. Also HEK293 cells transfected with BCRP variants, both wild-type (Arg at position 482, HEK293/R) and mutants (Gly or Thr at position 482, HEK293/G and HEK293/T), showed a markedly decreased imatinib accumulation, which could almost be completely reversed by the BCRP-specific inhibitor Ko143 (Burger *et al*, 2004). Also the specific BCRP inhibitor fumitremorgin C (FTC) could reverse the two- to three-fold resistance to imatinib of BCR-ABL-expressing cells transduced and selected to overexpress BCRP (K562/BCRP-MX10) (Nakanishi *et al*, 2006). Moreover, Brendel *et al* (2007) postulated that imatinib is a BCRP substrate based on the observations that (a) BCRP-transduced K562 cells were two- to three-fold resistant to imatinib-induced apoptosis and that inhibition of BCRP with FTC completely abrogated the resistant phenotype, (b) imatinib directly interacts with BCRP at the substrate binding site and stimulates BCRP ATPase activity, and finally (c) BCRP-transduced cells displayed significantly less imatinib accumulation. Although this study provides strong evidence for imatinib as a BCRP substrate, it may also point to the fact that imatinib transport by BCRP is concentration dependent since imatinib transport was facilitated only at low concentrations (<1 *μ*M). Confirmative experimental evidence for this notion was presented by Shukla *et al* (2007), who reported a narrow concentration range within which BCRP can transport TKIs and, in particular, imatinib. Thus, although the controversy may persist whether or not imatinib is a BCRP substrate, this hypothesis might help to explain the contradictory results, since different concentrations of the drug have been used in various literature reports.

**Figure 1:**
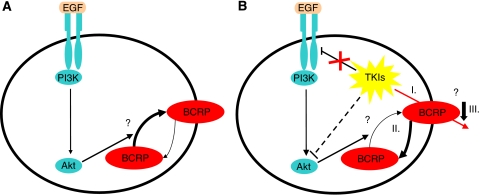
Interaction between TKIs and BCRP. An active PI3K–Akt pathway is apparently important for BCRP expression and localisation in the plasma membrane. (**A**) Stimulation of this pathway with EGF, for example, will phosphorylate Akt, leading to BCRP localisation to the plasma membrane. (**B**) (I) BCRP can actively efflux TKIs, thus inducing resistance to these drugs. However, BCRP-mediated TKIs resistance might be abrogated by TKIs inhibition of the PI3K–Akt pathway, which can lead to (II) BCRP relocalisation to the intracellular compartment and/or (III) decreased BCRP expression.

Other interactions besides being a possible substrate or inhibitor seem to exist, since imatinib itself could attenuate its resistance by suppressing BCRP expression (Nakanishi *et al*, 2006). Interestingly, however, imatinib decreased the expression of BCRP only in K562/BCRP-MX10 cells expressing BCR-ABL, but not in cells lacking BCR-ABL expression. The underlying mechanism for these differential responses involved downstream effects of imatinib inhibition of BCR-ABL, leading to the decreased phosphorylation of Akt, subsequently leading to reduced BCRP expression (Nakanishi *et al*, 2006). This study showed that an active PI3K–Akt pathway is responsible, at least in part, for maintenance of BCRP expression (Figure 1). Additionally, the PI3K–Akt signalling pathway may regulate the cellular localisation of this transporter. In this context, Mogi *et al* (2003) showed that Akt inhibition by LY294002 provoked translocation of Bcrp1 from the plasma membrane to the cytoplasmic compartment of side population (SP) cells.

Recent studies suggested that BCRP, along with P-gp, might limit the brain penetration of imatinib, reinforcing the idea that this TKI is a BCRP substrate. Breedveld *et al* (2005) showed that *Bcrp1* knockout mice displayed significantly increased imatinib brain penetration and decreased imatinib clearance compared with wild-type mice. Additionally, they have shown that co-administration of BCRP and P-gp inhibitors improved the brain penetration of the drug in wild-type mice. Similarly, Bihorel *et al* (2007) showed that blockade of both P-gp and Bcrp1 significantly increased the brain penetration of imatinib and its metabolites. Of note, however, the blood concentration and brain penetration of imatinib were unaltered in *Bcrp1* knockout and wild-type mice. The authors postulated that a functional P-gp activity in the blood–brain barrier of *Bcrp1* knockout mice might be dominantly responsible for retaining a similar brain uptake of imatinib as compared to wild-type animals.

### Nilotinib

Nilotinib is a novel BCR-ABL TKI, more potent and selective than imatinib. Brendel *et al* (2007) showed that BCRP-overexpressing K562 cells were two- to three-fold resistant to nilotinib; however, this was observed only at very low concentrations (10 and 25 nM), suggesting that resistance to nilotinib may not occur at clinically relevant concentrations. Notwithstanding these facts, the notion that nilotinib is a substrate for BCRP was supported by observations that it interacted with the BCRP substrate binding site, it stimulated the ATPase activity of this transporter and its accumulation was significantly suppressed in BCRP-transduced cells. Of further interest, nilotinib appeared to be a more potent inhibitor of BCRP than imatinib.

### Gefitinib

Contradictory data have been published also for the epidermal growth factor receptor (EGFR) TKI gefitinib, since some authors describe it as a BCRP substrate, while others classified it as an inhibitor and not a substrate (Elkind *et al*, 2005; Nakamura *et al*, 2005). Most likely, the apparent discrepancy in these results is due to the selected concentrations of gefitinib used in the different studies. Elkind *et al* (2005) showed that low concentrations of gefitinib (<1 *μ*M) significantly activated BCRP-ATPase activity in isolated membranes of BCRP-expressing mammalian MCF-7/MX and A431 cells, whereas higher concentrations (>1 *μ*M) had a markedly lower stimulatory effect. Consequently, this might explain the lack of gefitinib transport into vesicles of PC-6/SN2-5H cells, since a gefitinib concentration of 30 *μ*M was used in this study (Nakamura *et al*, 2005). These results suggest that, as discussed above for imatinib, gefitinib might also have a narrow window, especially in a low concentration range, where its active transport by BCRP is efficient. Consistent with gefitinib being a BCRP substrate, BCRP-transduced A431 cells were resistant to gefitinib compared with the parental cell line (Yanase *et al*, 2004; Elkind *et al*, 2005) and the resistant phenotype was reversed by the BCRP-specific inhibitor Ko143 (Elkind *et al*, 2005). Of note, consistent with EGFR amplification and dependence on EGFR signalling for survival, A431 cells are highly sensitive to gefitinib, with IC_50_ values in the nanomolar range. Conversely, K562 and P388 cells transduced with BCRP did not show gefitinib resistance (Yanase *et al*, 2004). In fact, wild-type K562 and P388 cells are relatively resistant to gefitinib with IC_50_ values in the 1–10 *μ*M range being compatible with their lack of inherent EGFR expression. Consistent with this, we have recently found that human EGFR-expressing Caco-2 colon carcinoma cells exhibit gefitinib IC_50_ values in the 300–600 nM range; in these cells, BCRP overexpression induced gefitinib resistance (Lemos *et al*, 2006a). In contrast, MCF-7/MR, with very low EGFR expression, displayed IC_50_ values for gefitinib in the 5–10 *μ*M range and in these cells BCRP overexpression was not a determinant of gefitinib resistance (data not shown). Thus, it has been hypothesised that BCRP is one of the determinants of gefitinib resistance in cells that express EGFR and show EGFR-dependent growth (Yanase *et al*, 2004). In fact, Elkind *et al* (2005) have shown that BCRP expression protects cells from gefitinib-mediated inhibition of EGFR phosphorylation and subsequent apoptosis. This suggests that BCRP prevents the action of gefitinib by effluxing this compound from the cell before it can interact with plasma membrane-associated EGFR. Similar to canertinib and imatinib, gefitinib is also a BCRP inhibitor and reverses BCRP-mediated drug resistance both *in vitro* and *in vivo* (Yanase *et al*, 2004; Nakamura *et al*, 2005).

### Erlotinib

Erlotinib is another EGFR TKI whose interaction with BCRP has been studied to a lesser extent. Nonetheless, a preliminary study suggests that erlotinib is also a substrate of BCRP (van Tellingen *et al*, 2007). Since both gefitinib and erlotinib can affect phosphorylation of Akt, downstream of EGFR, this mechanism may also be involved.

## BCRP AND TKI BIOAVAILABILITY

It has been shown that BCRP is highly expressed in the small intestine and colon, suggesting that this transporter is involved in the regulation of uptake of substrates from the gastrointestinal tract (Maliepaard *et al*, 2001). BCRP may mediate the transport of substrates from mucosal cells back to lumen. Since all discussed TKIs are orally active compounds, BCRP might limit their activity not only by mediating extrusion and inducing resistance in the tumour cells, but also by reducing their oral bioavailability. Using Caco-2 cells as an *in vitro* model for intestinal drug transport, Burger *et al* (2005) found that continuous exposure to imatinib upregulates the expression of BCRP and P-gp, which resulted in a decreased intracellular accumulation of imatinib. We have also recently shown that BCRP expression in Caco-2 cells is upregulated under folate-deficient conditions (Lemos *et al*, 2006b). Folates might also affect the subcellular localisation of BCRP (Ifergan *et al*, 2005). Thus, modulation of expression of BCRP and other ABC transporters in the gut might have a crucial role in the bioavailability of orally administered drugs.

## BCRP VARIANTS

Disparities in the transport of rhodamine 123 among BCRP-overexpressing cell lines led to the identification of a mutational hot spot located in codon 482 where a single amino-acid change had occurred (Robey *et al*, 2007). Wild-type BCRP, with an arginine at position 482 (R482), facilitated efficient transport of mitoxantrone, but not rhodamine 123 or doxorubicin. In contrast, cells carrying a glycine (R482G) or a threonine (R482T) at position 482 were able to transport rhodamine 123 and doxorubicin, while also maintaining their ability to transport mitoxantrone. The BCRP variants were found in drug-resistant S1-M1-80 (R482G) and MCF-7 AdVp3000 (R482T) but not in the parental S1 and MCF-7 cell lines, suggesting that these were acquired mutations resulting from drug selection. Inside-outside vesicles from these cell lines do not influx methotrexate. Hence these R482G cells were not resistant compared to cells with wild-type BCRP when continuously exposed to methotrexate (Chen *et al*, 2003). However, when a short 4 h exposure was used, the mutant R482G was highly resistant to methotrexate (Assaraf, 2006). This discrepancy can possibly be explained by the use of vesicles in which only uptake of native methotrexate was measured, while in intact cells, continuous exposure to methotrexate leads to massive polyglutamylation, preventing efflux in both wild-type and mutant cells. At a short exposure, polyglutamylation is less extensive, leading to efflux of the parent drug and lower polyglutamylates. Such studies show that vesicles are excellent models to study transport, but do not provide an insight into the role of cellular metabolism and transport.

It is now clear that BCRP mutations have a high impact on the substrate specificity of this transporter and that cells harbouring these mutations are cross-resistant to a wider variety of chemotherapeutic agents (Assaraf, 2006). However, BCRP mutations at amino acid 482 have not yet been found in clinical samples, suggesting that their relevance for clinical drug resistance may be limited.

Several single nucleotide polymorphisms (SNP) in the *ABCG2* gene have been reported that might have an important impact on BCRP protein expression and function (Table 1). A nonsynonymous SNP C421A resulting in a glutamine to lysine amino-acid change at position 141 (Q141K) has been associated with markedly decreased levels of BCRP protein expression and also low levels of drug resistance. Nonetheless, cross-resistance patterns were similar for the wild type and the Q141K BCRP variant, suggesting that this polymorphism does not affect substrate recognition of BCRP (Imai *et al*, 2002). More recently, Cusatis *et al* (2006) reported that the C421A polymorphism was statistically significantly associated with the occurrence of diarrhoea in a cohort of 173 Caucasian patients with non-small-cell lung cancer receiving treatment with oral gefitinib. In the group of 16 patients carrying at least one A421 allele, 7 (44%) developed diarrhoea, while only 12 out of 108 (12%) patients had diarrhoea among the group carrying the wild-type genotype. The reduced protein levels and altered ATPase activity of the BCRP C421A variant might affect the oral absorption and/or elimination pathways of gefitinib and thereby increase the steady-state gefitinib plasma concentrations leading to diarrhoea. In contrast, Gardner *et al* (2006) did not find significant differences in the pharmacokinetic parameters of imatinib *in vivo* between 16 patients heterozygous for the C421A SNP compared with 66 patients harbouring the wild-type sequence. This result was unexpected, since the authors showed that HEK293 cells transfected with wild-type BCRP accumulated significantly less imatinib than HEK293 cells transfected with the C421A BCRP variant, despite similar levels of protein expression. In a Japanese population, 57 out of 124 patients expressed the A421 allele, indicating that the A421 allele frequency in this population is quite high (Imai *et al*, 2002). The C421A variant seems less frequent in other ethnic groups (Yanase *et al*, 2006). Kondo *et al* (2004) also observed that the C421A SNP was associated with a 60–70% lower protein expression compared to that of the wild-type. Another SNP, G1322A (S441N), showed even lower levels of protein expression. Contrary to the wild-type and all other BCRP variants that had an apical membrane localisation, this latter BCRP variant was expressed intracellularly, suggesting that BCRP SNPs might also affect its cellular localisation. Another SNP, the C376T, replaces glutamine by a stop codon at position 126 (Q126T), and was found in 3 out of the 124 Japanese population (Imai *et al*, 2002). Despite its low frequency, this C376T polymorphism may have a higher impact than the C421A polymorphism because no active BCRP protein can be expressed from this gene. Finally, another BCRP SNP found in this Japanese population, G34A, which replaces valine by methionine at position 12 (V12M), showed similar protein expression and drug resistance levels as the wild-type (Imai *et al*, 2002).

From these studies, it is clear that BCRP expression and localisation are regulated at different levels, in which various polymorphisms seem to play an important role.

## BCRP IN STEM CELLS

Stem cells have the unique ability of self-renewal and can differentiate into a variety of specialised cell types. The SP phenotype, which is characterised by the ability to transport the fluorescent dye Hoechst 33342, has been identified as a characteristic feature of haematopoietic stem cells.

Owing to the ability of P-gp to transport Hoechst, initially this transporter was thought to be responsible for the SP phenotype. However, since mice deficient in P-gp had a normal number of bone marrow SP cells, it was concluded that P-gp is not required for the SP phenotype. A more detailed examination of other ABC transporters in bone marrow cells of P-gp knockout mice revealed high levels of Bcrp1 mRNA and distinct levels of Mrp1, Mrp3 and Mrp4 mRNA (Zhou *et al*, 2001). The notion that only BCRP can efflux Hoechst demonstrated that this transporter was dominantly associated with the SP phenotype, which was further confirmed by BCRP transfection in bone marrow cells causing significant expansion of cells bearing the SP phenotype (Zhou *et al*, 2001). Also in Bcrp1/Mdr1a/1b triple knockout mice, it was shown that Bcrp1, but not Mdr1a/1b, is responsible for the SP phenotype in the bone marrow, while both transporters are required for the SP phenotype in the mammary gland (Jonker *et al*, 2005). It has now been demonstrated that SP cells are present in several tumour samples, possess stem cell-like properties, overexpress BCRP and possess inherent drug resistance (Haraguchi *et al*, 2006). Consistently, BCRP overexpression was reported on primary CML CD34+ cells (Jordanides *et al*, 2006). Imatinib is efficiently used in the clinical treatment of BCR-ABL-driven CML, but possible BCRP-mediated imatinib resistance would naturally affect the efficacy of the treatment. Thus, targeting BCRP in these tumour stem cells with potent and specific inhibitors might constitute an important strategy to eliminate or reduce the tumour stem cell population and improve the efficacy of targeted therapy.

## CONCLUSION

Overexpression of BCRP can provoke MDR in cancer cells, although its role in clinical drug resistance remains unclear (Robey *et al*, 2007). Several TKIs have recently been shown to interact with BCRP; thus, BCRP can confer resistance to these drugs, both at the tumour cell level and by decreasing their oral bioavailability. A hot spot mutation at amino acid 482 and several SNPs have been described for the BCRP gene, which may affect substrate specificity, expression and function of this transporter. These genetic variations must, therefore, be taken into account when targeting BCRP to reverse/modulate drug resistance. The expression of BCRP in SP cells, and in particular SP of tumour cells, might also be of great interest in the development of new anticancer strategies.

## Figures and Tables

**Table 1:** BCRP substrates and polymorphisms

***Substrates***		
**Classical anticancer drugs**	**Novel targeted drugs**	
Mitoxantrone	Canertinib (CI-1033)^a^	
Anthracyclines^b^	Imatinib^a^	
Camptothecins	Nilotinib^a^	
Antifolates^b^	Gefitinib^a^	
	Erlotinib	
	Flavopiridol	
		

***Polymorphisms*** c		
**Variant**	**Amino-acid change**	**Effect**
G34A	Val12Met (V12M)	No change
C376T	Gln126stop (Q126T)	No active BCRP protein
C421A	Gln141Lys (Q141K)	Decreased protein levels and drug resistance
		Increased gefitinib-associated toxicity (diarrhoea)
		Increased imatinib accumulation *in vitro*, but no changes in the pharmacokinetic parameters of imatinib *in vivo*
G1322A	Ser441Asn (S441N)	Decreased protein levels and different subcellular localisation

BCRP=breast cancer resistance protein.

a Compounds that are simultaneously substrates and inhibitors.

b Efflux of compounds affected by mutation at position 482.

c More BCRP SNPs have been described, but no relation with functional activity or protein expression was reported (summarised by Yanase *et al*, 2006).
